# Combating Infectious Diseases with Synthetic Biology

**DOI:** 10.1021/acssynbio.1c00576

**Published:** 2022-01-25

**Authors:** Anooshay Khan, Julian Ostaku, Ebru Aras, Urartu Ozgur Safak Seker

**Affiliations:** † UNAM − National Nanotechnology Research Center, Institute of Materials Science and Nanotechnology Bilkent University, 06800 Ankara, Turkey

**Keywords:** synthetic biology, infectious diseases, engineered phage and bacteria, SARS-CoV-2, diagnostics, therapeutics

## Abstract

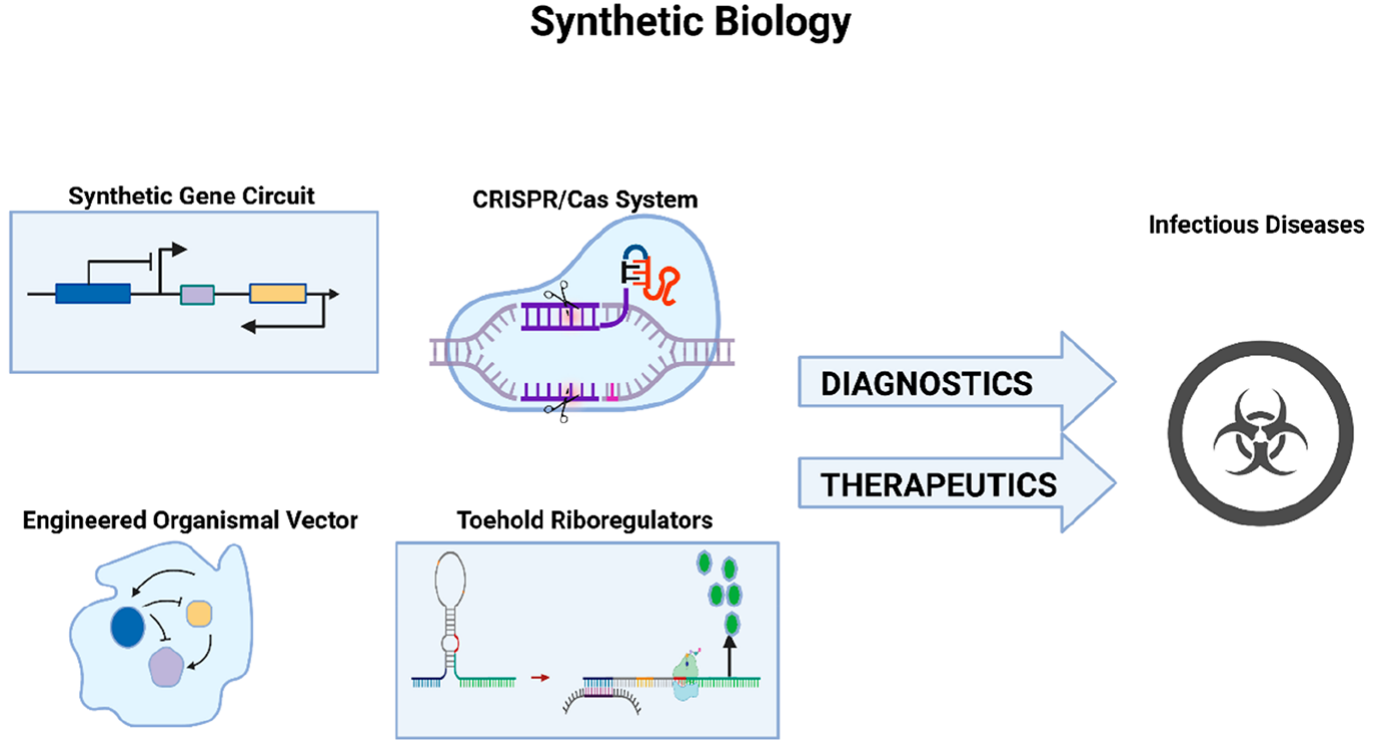

Over the past decades, there have been numerous outbreaks, including parasitic, fungal, bacterial, and viral infections, worldwide. The rate at which infectious diseases are emerging is disproportionate to the rate of development for new strategies that could combat them. Therefore, there is an increasing demand to develop novel, specific, sensitive, and effective methods for infectious disease diagnosis and treatment. Designed synthetic systems and devices are becoming powerful tools to treat human diseases. The advancement in synthetic biology offers efficient, accurate, and cost-effective platforms for detecting and preventing infectious diseases. Herein we focus on the latest state of living theranostics and its implications.

Throughout history, infectious diseases have caused havoc in every stage of civilization. From causing economic distress to the complete breakdown of societies, infectious diseases are a defining part of the human tale. For instance, the Ebola epidemic in West Africa (2013–2016) affected the already poor healthcare system owing to the high number of reported cases.^1^ Similarly, the spread of SARS from 2002 to 2003 affected economic sustainability, particularly in Canada and Singapore, due to limited trade and travel.^2^ Most recently, the current COVID-19 pandemic has disrupted entire socioeconomic structures globally, pushing biologists in their mission to tackle the issue through new and innovative solutions.

Since the discovery of penicillin in 1928, antibiotics have remained the leading treatment choice for most bacterial infections. Its discovery marked the beginning of an era where natural product antibiotics were researched and developed.^3^ This continued until the mid-1950s, when research for new antibiotics drastically declined and has since remained in the same debilitated state.^3^ Due to their high efficacy, antibiotics have shown remarkable progress and improvements in drastically lowering the number of deaths caused by bacterial infections. However, alongside the discovery and use of antibiotics, antibiotic-resistant bacteria are also on the rise. The rapid rate at which bacteria are evolving is disproportionate to the rate of development for new antibiotics, which aim to potentially combat the novel infections resulting from the bacteria. Moreover, the fact that existing antibiotics are incapable of distinguishing between pathogenic bacteria and our microbiota, warrants the need for research into using alternatively effective methods that could provide specificity.

Another major problem faced while combating infectious diseases, especially the viral infectious diseases, results from the available diagnostic methods, where conventional testing methods tend to consume a significant amount of time and lack of accuracy. The two main diagnostic techniques used nowadays, RT-qPCR and ELISA, are incompatible with the existing infrastructure of available point-of-care (POC) testing. More time, money, and resources are now required to ensure that laboratories are compatible with clinics, hospitals, and other healthcare facilities to enable effective diagnosis.^4^ These issues have resulted in a heightened interest in synthetic biology and the development of strategies associated with living therapeutics.

Synthetic biology is a field of science aimed at creating new biological parts that have specific functions or redesigning the existing ones, granting them a new function. The most compelling promise of synthetic biology as a solution to biomedical challenges is the engineering of microorganisms capable of detecting pathogens, delivering therapeutic agents, and controlling the dosage required to meet safety concerns. In this review, we focus on the latest state of both living therapeutics and diagnostics, their implications, and the associated challenges. We covered the latest developments in the field generally in the past five years. Also we have discussed the impact of synthetic biology on the COVID-19 pandemic.

## Using Bacteria to Detect and Attack Infectious Diseases

Synthetic biology focuses on reprogramming cellular senses and their responses by engineering genetically modified biological systems that can perform novel functions.^5^ While the advancements in synthetic biology offer robust, inexpensive, and rapid platforms for detecting and eradicating diseases, four main steps should be considered during bacteria engineering in the fight against infectious diseases.^6^

**Figure 1:**
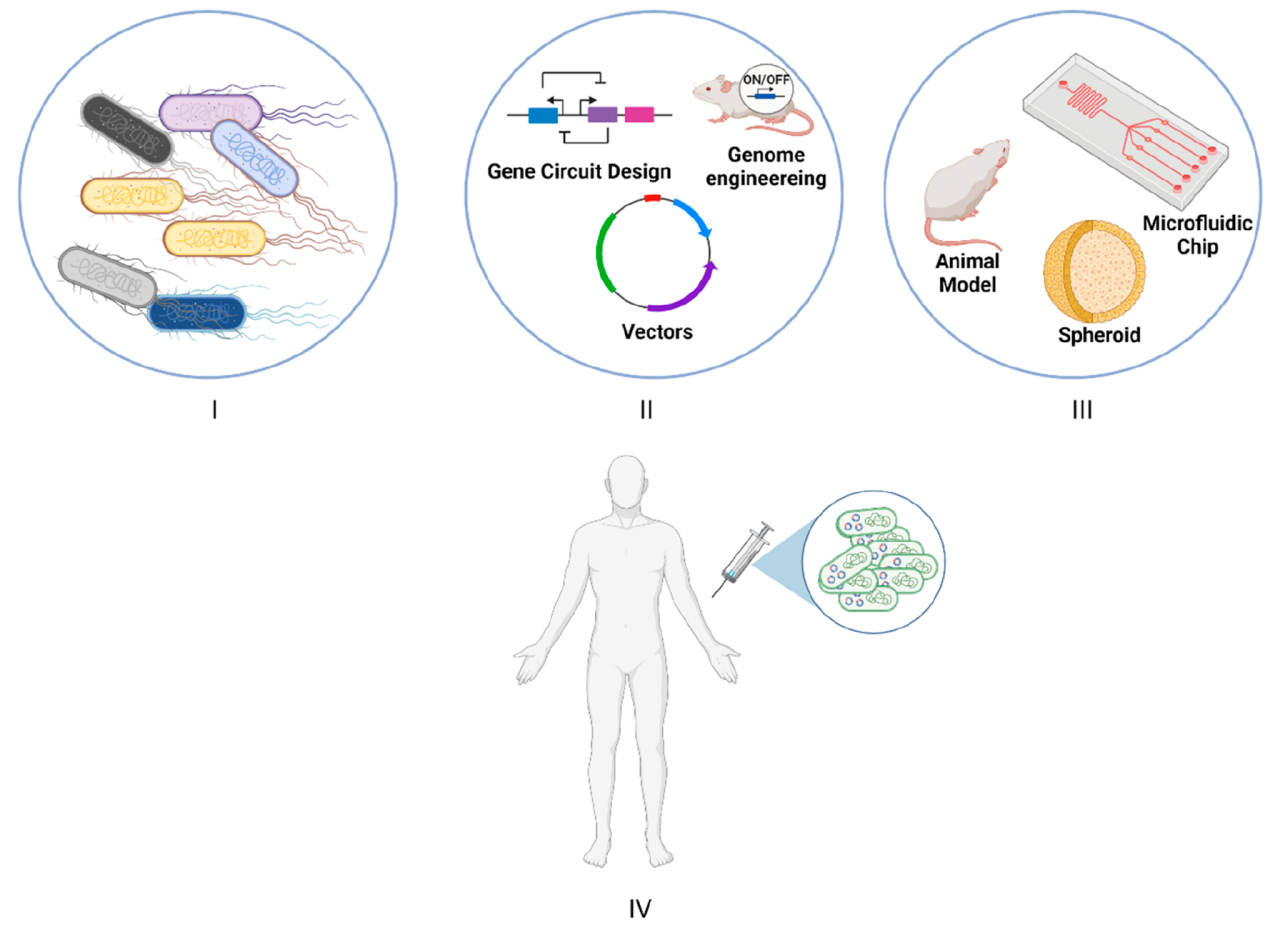
Schematic representation of the workflow in bacteria based living therapeutics. (I) Selection of the organism to be used. (II) Engineering living therapeutics. Genome engineering and/or plasmid with optimized and efficient genetic circuits can be implemented. (III) Testing the systems in vitro via mammalian cell culturing, spheroids, or microfluidic chips or in vivo using model organisms. (IV) Human trial.

As summarized in Figure 1, the first step is the selection of bacteria that will be engineered. The human microbiota is populated by 1000 bacterial species, also known as commensal bacteria, which play an essential role in the well-being of hosts.^7^ Their dysregulated interaction with the host organism has been shown to correlate with various diseases such as obesity, cancer, inflammatory bowel syndrome (IBD), and many more.^8,9^ Due to these reasons and the fact that foreign bacteria would trigger an immune response, commensal or attenuated bacteria selection is crucial.^10−12^ The second step is genetic circuit design which should mediate the production of the therapeutic agent and it is in situ administration. Regarding the therapeutic agent, different toxins, peptides, or proteins that arrest the growth of the infectious agent or eliminate it at all can be selected.^13^ Moreover, the delivering method should be considered, which involves intracellular production of the drug agent by bacteria and then secretion to the extracellular space by different secretion systems or bursting of the cell and release of the agent.^14^ When designing the genetic circuit, it is important to mediate the desired output (therapeutic element), upon administration of different chemical or physical outputs (infectious bacteria). Lately, there has been an increased interest in developing genetic circuits with tunable detection thresholds which would tune and increase the performance and reliability of the outputs at desired levels.^15,16^ Synthetic gene regulatory circuits that are able to improve the fold change activation of a target promoter have also been designed and can serve as a helpful tool when engineering living therapeutics.^15−17^ In order to control bacteria growth at the specific target tissue, metabolic auxotrophy can be used. Also, a recent study by Chien et al. showed a design of different biosensors that can control the growth of the bacteria at a specific organ based on pH, lactate, and oxygen signatures of the organ’s microenvironment.^18^ Lastly, the administration of bacteria upon drug delivery is critical. Upon achieving the goal, the bacteria should be eradicated from the body. To do so, there are different solutions: (a) the bacteria can be engineered to have no antibiotic resistance so that antibiotics can be administered to rid of the therapeutic bacteria upon finalization of the therapy or (b) a synchronized lysis circuit can be integrated into the bacteria’s genome, which would cause bacterial population lyses once a critical population density is achieved.^19,20^ The latter would cause an effective release of the cargo as well as total elimination of the bacteria.

In order to test the bacteria, traditional in vitro and in vivo experimental procedures can be followed, or microfluidic chips that would mimic the microenvironment of the targeted organ can be used. In that regard, Harimoto et al. built a platform able to monitor engineered bacteria in multicellular spheroids, and by this, they aim to accelerate clinical applications for synthetic biology.^21^

**Figure 2:**
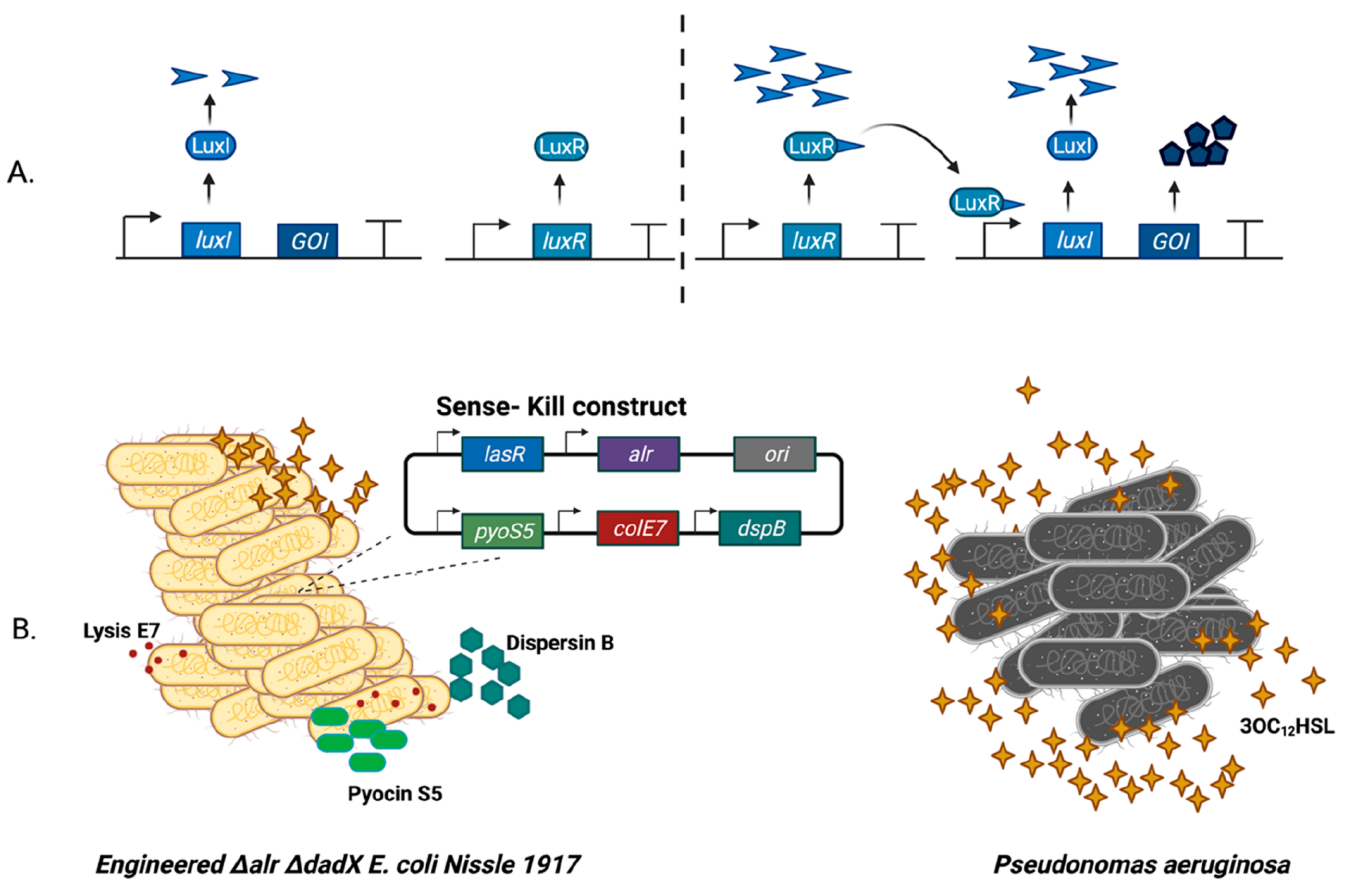
(A) Schematic representation of quorum sensing working principle. The left panel shows the basal level expression of both *luxI* and *luxR* genes due to low cell densities of bacteria. *luxI* gene produces the autoinducer protein, which will diffuse to the outer surface but will not be able to activate transcription of *lux* box. On the right, a high number of bacteria is present; hence more autoinducers will be diffused to the outer surface transcription of *lux* box where the GOI (gene of interest) is located will be mediated. (B) Engineered *Δalr ΔdadXE. coli* Nissle 1917 to sense and kill *P. aeruginosa* via 3OC_12_HSL (quorum-sensing molecule released by *P. aeruginosa*). Upon detection of the infection site via 3OC_12_HSL the engineered cells will induce their own lysis via Lysin E7 and release Dispersin B and Pyocin S5, both effective in treating *P. aeruginosa*.^30^

By engineering commensal bacteria, researchers have successfully constructed living whole-cell biosensors with robust genetic circuits that precisely detect and attack infectious agents and their related pathologies. Such a probiotic-based diagnostic system was reported for cholera by Mao et al.^22^ Cholera is an acute diarrheic disease caused by the infectious agent *Vibrio cholerae*, and according to World Health Organization (WHO) it is the cause of death for 525 000 children under five years old every year.^23^ In their design, Mao et al. engineered a *Lactococcus lactis* strain that could detect *V. cholerae* via its specific quorum-sensing autoinducer molecule (CAI-1).^22^ Quorum sensing is a process by which bacteria modulate their gene expression in response to the concentration of a self-produced autoinducer (AI) (Figure 2A); it is considered a social behavior of bacteria in which populations undergo mutual changes that mediate the expression of genes that help bacteria thrive at high cell densities.^24^*V. cholerae* produces CAI-1 and autoinducer 2 (AI-2), but only CAI-1 is specific to genius *Vibrio*; therefore, Mao et al. built a two-component hybrid receptor that consisted of the binding domain of CAI-1 and expressed it in *L. lactis*, a commensal bacterium.^22^ By using this system, they were able to detect the presence of *V. cholerae* in mice by analyzing their fecal samples. Moreover, Holowko et al. engineered a synthetic sensing system in nonpathogenic *Escherichia coli* based on CAI-1 quorum sensing of *V. cholerae*.^25^ In this design, the researchers created a synthetic genetic sensing system comprising of CqsS, LuxU, and LuxO proteins in *E. coli* which enabled precise detection of *V. cholerae* quorum sensing molecules. A green fluorescence protein (GFP) was constructed under the pQrr4 promoter, which is downregulated in the presence of CAI-1. Furthermore, the sensor was conjugated with a clustered regularly interspaced short palindromic repeat (CRISPR) based inverter. In the presence of the autoinducer CAI-1, the CqsS sensory machinery activated a downstream signaling cascade, which in turn down-regulated gRNA. The latter repressed CRISPRi activity, ultimately leading to the expression of a reporter GFP only in the presence of *Vibrio* derived CAI-1.^25^ Using this system, they were able to sense the presence of *V. cholerae* supernatant. In a later study the authors repurposed their system to sense and kill *V. cholerae*.^26^ They coupled Art-085, and YebF-Art-085 to their existing biosensing mechanism. Art-085 was used as a therapeutic agent to kill the infectious bacteria, whereas YebF-Art-085 (YebF is a protein secretion tag that directs the protein localization in bacteria periplasm) mediated cell lysis required for the proper release of Art-085 to the outer surface. Upon detection of *V. cholerae*, by the sense and kill mechanism, YebF-Art-085 is expressed and localized to *E. coli*’ s periplasma. The fusion protein punctures the outer membrane, and therefore, the constitutively produced protein, Art85, is released to the cell medium and eliminates *V. cholerae*. By using this system, the authors were able to inhibit the growth of *V. cholerae* cell effectively.

Furthermore, *E. coli* strains have also been engineered for the precise elimination of *Pseudomonas aeruginosa.* This bacterium is a human pathogen, which colonizes the respiratory and gastrointestinal tract, and is one of the most problematic hospital-acquired infections due to the increase in number of its antibiotic resistant strain attributed mostly to biofilm formation.^27,28^ Saeidi et al. engineered a pathogen sensing and killing system in *E. coli* based on detecting acyl-homoserine lactone (AHL), a quorum sensing molecule produced by *P. aeruginosa*.^29^ In their study they were able to build a system that can sense the AHL molecules produced by *P. aeruginosa*, and produce pyocin S5, a bacteriocin, as a response alongside E7 lysis protein that would mediate the bursting of the cells and release of pyocin S5. Using their design, they were able to repress biofilm formation close to 90%, and when testing their cells in planktonic *P. aeruginosa* they were able to reduce viability up to 99%. Advancing on this foundation, the group generated an improved version of the system, this time using Δ*alr* Δ*dadXE. coli* Nissle 1917, a nonpathogenic probiotic strain, as a host (Figure 2B).^30^ The used bacteria lacked *alr* and *dadX* genes, which play a role in d-alanine metabolism. The latter is a building block of peptidoglycan in Gram-negative bacteria, therefore limiting its growth only in the presence of a supporting plasmid encoding for these genes. By adding this gene to their genetic system, the authors were able to eliminate the usage of an antibiotic selection marker which would risk horizontal gene transfer of the antibiotic resistance gene to other bacteria. In addition, dispersin B (DspB), an antibiofilm protein, was added into their designed genetic system to help disrupt mature biofilms and mediate a better therapy. When testing their system in vivo, they were able to show therapeutic and prophylactic activity in both *Caenorhabditis elegans* and *Mus musculus*.

In another recent study, a genome-reduced *Mycoplasma pneumoniae* (namely CV2, lacking *mpn372* and *mpn133* genes) was engineered and tested for treatment of biofilm formation from *Staphylococcus aureus*.^11^ Garrido et al. first attenuated the bacterium to mediate its in vivo application and tested it in catheter-associated biofilms. Therapeutic elements dispersin B (DspB) and lysostaphin, a bacteriocin shown to be primarily active against methicillin-resistant *S. aureus*,^31,32^ were introduced into the attenuated strain via a gene platform. To mediate efficient secretion of the therapeutic elements, they identified and optimized a secretion signal, *mnp140Opt*, within their attenuated strain. The secretion tag was fused to both DspB and lysostaphin and shown to improve protein production and secretion levels. The authors tested CV2 bacteria expressing DspB and CV2 bacteria expressing both DspB and lysostaphin in vivo. They observed a better activity when administering CV2-DspB-Lysostaphin compared to CV2-DspB, which showed no efficacy in dissolving catheter-associated biofilms. When compared to wild type *M. pneumoniae*, CV2-DspB-Lysostaphin showed lower efficacy, which concludes that more improvements can be done, but the results are promising.

## Engineered Phages for the Detection and Treatment of Infectious Diseases

Virulent bacteriophages are viruses that infect and kill bacteria. They do so by attaching to a specific receptor on the host cell surface, releasing their genomic content inside. The bacteriophage then replicates within the bacterium, releasing hundreds of progeny bacteriophages and lysing the bacteria in the process.^33^ Ever since their discovery as therapeutic agents against *Shigella dysenteriae* in 1919, bacteriophages have been explored to treat bacterial infections. Phage therapy has been successful against several bacterial infections such as cholera, diabetic foot ulcer, typhoid, and chronic otitis; however, the limitations of using phage-based systems arise from their failure to penetrate the cell walls, the limited phage-host range, and increase in the bacterial resistance.^34−38^

**Figure 3:**
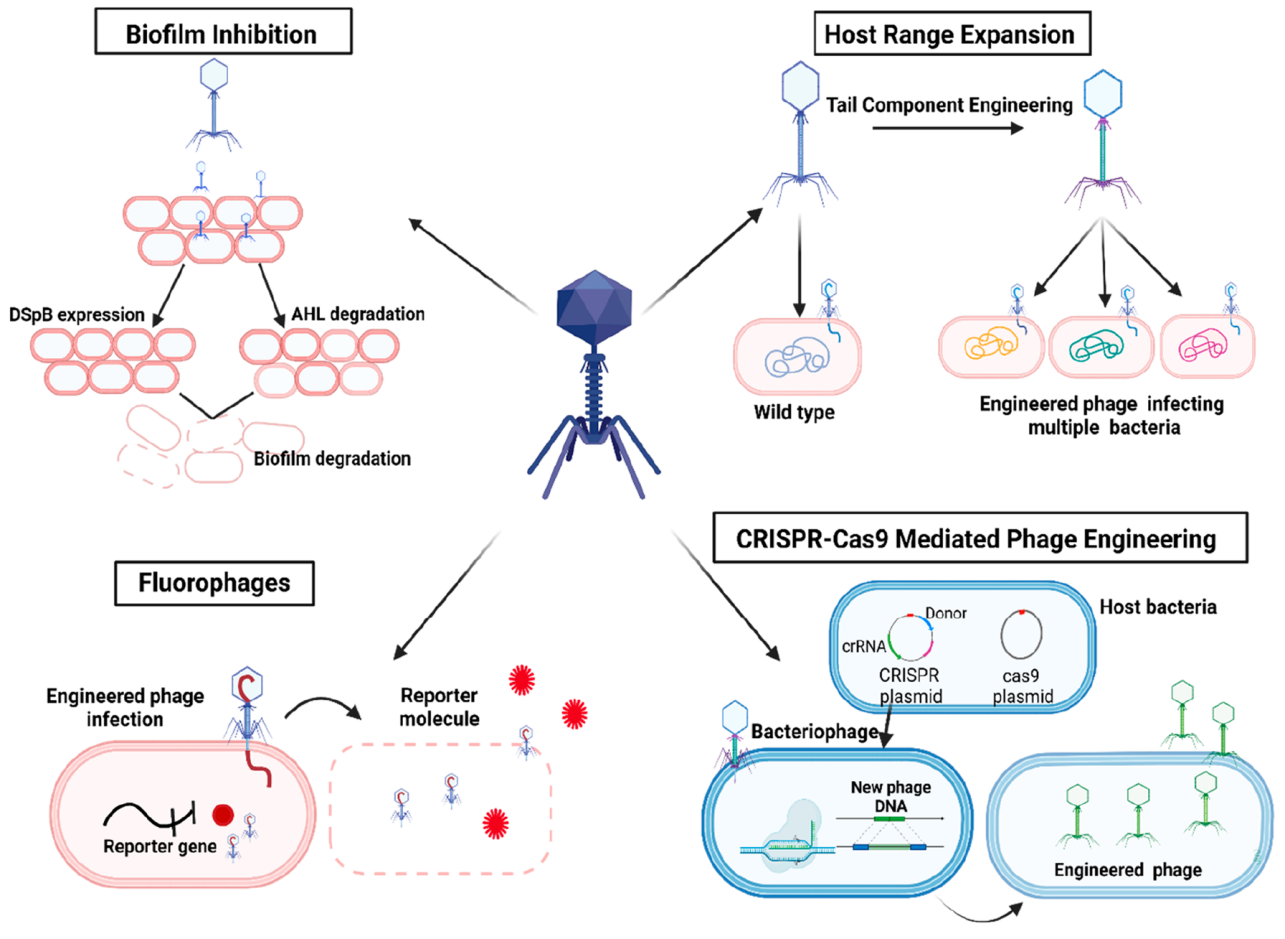
Schematics summarizing various ways by which phages can be engineered to overcome the limitations of phage-based treatments and diagnosis of infectious diseases.

As shown in Figure 3, synthetic biology offers solutions to the aforementioned limitations of phage therapy by engineering phages, thus making them safer and effective for therapeutic purposes. Phages invade bacteria by attaching their tails to the host cell surface, where the tail component can recognize a specific host strain.^33^ Phages have a defined host range that is dependent on several endogenous and exogenous host factors. These include the phage receptors, the defense mechanism of the bacteria, the receptor binding proteins, and environmental conditions. Through synthetic biology, the tail component of phages can be engineered to recognize multiple host strains, thereby expanding the host range. Ando et al. reported a yeast-based phage engineering system to modulate the *E. coli* phage (T7, T3) host range by engineering phage tail components, targeting Yersinia and Klebsiella bacteria.^39^ These engineered phages then showed enhanced killing activity against the new bacterial target.^39^ Inspired by antibody specificity engineering, Yehl et al. identified and genetically modified the host-range-determining regions (HRDRs) in the T3 phage tail fiber to produce synthetic “phagebodies”. Following the site-directed mutagenesis, these phagebodies were able to retain the overall tail fiber structure, showed alteration in host ranges, target resistant bacterial mutants.^40^ The use of the prokaryotic adaptive immunity systems CRISPR-Cas system has revolutionized gene editing and genetic engineering in the detection and diagnosis of diseases. CRISPR/Cas9 system can be integrated into the genome of temperate phages to enhance their efficiency. By using this strategy, Park et al. developed a phage-based CRISPR/Cas9 delivery system by modifying the genome of a temperate phage ϕSaBov.^41^ The modified phages generated by removal of viral content demonstrated an enhanced *S. aureus* specific-killing in both in vitro and in vivo experiments. The findings of this study laid solid grounds for the development of CRISPR/Cas9 antimicrobial specific to *S. aureus* infections.^41^ Initially, this system has shown promising results against external *S. aureus* infections. Cobb et al. evaluated the efficacy of this system against internal osteomyelitis and contiguous soft tissue infection in the murine model. Using a biofilm-forming strain of *S. aureus*, the researchers showed that CRISPR-Cas9 modified phages successfully mitigated bacterial infection in contrast to the unmodified phage.^42^

Biofilm production plays a significant role in the pathogenesis of a disease by making the bacteria resilient to the immune system and drug treatment. In addition to expanding the host range, phages have also been engineered to inhibit biofilm production by either expressing biofilm matrix-degrading enzymes or by inhibiting quorum sensing between bacteria, which subsequently results in biofilm inhibition. Recently, Landlinger et al. investigated endolysins PM-477 of the type 1,4-beta-*N*-acetylmuramidase encoded on *Gardnerella* prophages as a treatment for bacterial vaginosis.^43^ The study showed that by domain shuffling, several engineered phage-derived endolysins were able to completely disrupt the biofilm produced by *Gardnerella* bacteria during infection.^43^ Quorum sensing is a phenomenon in which bacteria communicate and regulate biofilm formation. Acyl-homoserine lactones (AHL) are the main component of quorum sensing which regulates this cellular signaling, and lactonase is well-known for its role as a quenching molecule in quorum sensing.^44^ Researchers have engineered quorum-quenching phages to inhibit biofilm production. For this purpose, the T7 bacteriophage was engineered to express the AiiA lactonase enzyme upon infection. T7 phage expressing the AiiA lactonase effectively degraded AHLs from the bacteria, inhibiting the biofilm production.^44^

Owing to their ability to form plaques from postbacterial infections, phages have also been used for the detection of bacterial infections.^45^ The advances in genome engineering and synthetic biology have enabled reporter genes to be incorporated into the phage genome, making them excellent candidates for the detection of infectious diseases. Rondon et al. reported a fluoromycobacteriophage, a reporter phage engineered to express fluorescent reporter genes, to detect *Mycobacterium tuberculosis*.^46^ In this study, the researchers engineered and optimized mycobacteriophage (mCherry-bomb) to express the mCherry-bomb gene upon detection of viable *M. tuberculosis* in patients’ sputum samples. In addition to this, the reporter phage was also able to determine Rifampicin resistance from the sputum sample.^46^ Phage-based diagnostics have made it possible for easy detection of infectious diseases with readable outputs. They are cost-effective, yield specific results, and are less time-consuming compared to conventional diagnostics methods, such as ELISA, CFT, PCR.^47,48^ The major drawbacks of phage-based diagnosis are the need for phage to infect bacteria and the possibility of false negatives. However, constant efforts are made to circumvent these limitations by advancements in molecular and genome engineering tools. Such bacterial and phage-based biosensors, which harness disease-specific biomarkers and produce specific and quantitative responses, have paved a way toward the next generation of medical diagnostics.

Phage-based prophylactic vaccines against infectious agents can reduce mortality and morbidity during endemics and pandemics. The ease of engineering phage genomes and their ability to infect bacteria make phages ideal for vaccination against infectious diseases. Deng et al. reported a tripartite live oral vaccine against influenza A infection.^49^ In their novel design, the researchers engineered a nonlytic bacteriophage f88 to display an influenza A virus epitope (matrix protein 2 ectodomain). These phages were able to infect the *E. coli* in the gut. Furthermore, *E. coli* cells were also engineered to express *Y. pseudotuberculosis*-derived invasin, which facilitated adhesion to the gut mucosa. When administered orally as a live bacterium-phage combination, the engineered gut-colonizing *E. coli* were able to produce these phages continuously. This allowed for long-term colonization of bacteria in the gut and prolonged the production of phages displaying viral epitope, resulting in an enhanced immunization and protection against influenza A virus infection.^49^ In addition to their ability to infect bacterial cells, phages can present molecules on their surface and elicit specific immune responses.^50^ Owing to this property, viral-like particles (VLPs) can be engineered to express specific epitopes on viral coat surfaces to provide vaccination.^50^ Tao et al. engineered Bacteriophage T4 for a dual vaccine against anthrax and plague simultaneously.^51^ This was achieved by displaying the *Bacillus anthracis* and *Yersinia pestis*. *Y. pestis* antigens on T4 small outer capsid protein. The engineered VLPs elicited specific immune protection against both anthrax and plague when administered in animal models.^51^ In a similar study, bacteriophage VLP was developed for Zika Virus (ZIKV) vaccination. Basu et al. demonstrated in vitro neutralization of the ZIKV by producing antibodies against different engineered phage VLPs, which presented ZIKV B cell epitopes.^52^ Under natural circumstances, bacteriophages can only infect bacterial cells; however, phages can also be engineered to penetrate mammalian cells.^53^ By exploiting this possibility, phage-based DNA vaccines were developed.^54^ In such systems, antigens are cloned in the nonessential regions of the bacteriophage genome that are placed under the control of a eukaryotic promoter. When mammalian cells are infected with these engineered phage particles, these particles act as DNA vaccines.^54^ Upon infections, these phage particles transcribe the antigen and present the antigens on the anaphase-promoting complexes (APCs), inducing a potent immune response.^54−56^ Bacteriophage capsids have sophisticated 3-D structures which are stable, can readily self-assemble, and can be engineered for packaging and delivering molecules in the body. These properties make bacteriophages great nanocarriers for drug delivery. RNA phage MS2 VLP devoid of viral genetic material has been thoroughly investigated for carrying antimicrobial cargos such as RNAs, DNAs, epitope peptides for combating infectious diseases.^57^ The precise detection of disease signals by bacteriophages allows the production of specific therapeutic molecules. These pioneering studies offer great potential for synthetic biology-inspired therapies to provide novel therapeutic strategies for future clinical applications.

## Synthetic Biology in the Era of Pandemic: SARS-CoV-2

### Synthetic Biology-Based Diagnostics and Therapeutics

Since December 2019, a newly identified coronavirus, named severe acute respiratory syndrome coronavirus 2 (SARS-CoV-2), causing severe pneumonia and acute, lethal lung failure, has rapidly spread first through China and then the rest of the world and developed into a pandemic. The scientific world has focused on rapid diagnostics and preventive vaccine and therapeutics development, by coordinating the use of biological data and bioengineering techniques, as coronavirus disease 2019 (COVID-19) continues to spread and to claim lives worldwide, 232 million afflicted people and almost 5 million deaths according to WHO, as of September 21, 2021.^58^

One essential step in addressing the threats of new and lethal pathogens is to generate rapid and reliable diagnostics tools. Synthetic biology techniques focusing on gene circuit constructions and novel biosensing systems that are capable of processing the inputs have been successfully shown to be an option compared to current conventional diagnostic tools.^59−64^

Various novel CRISPR-Cas-based diagnostics platforms, namely specific high sensitivity enzymatic reporter unlocking (SHERLOCK), 1 h low-cost multipurpose highly efficient system (HOLMES), or DNA endonuclease-targeted CRISPR trans reporter (DETECTR) systems have been devised to effectively detect biomarkers of the diseases.^65−67^ The methods rely mainly on identifying a certain target sequence related to the disease, like envelope (E) and nucleoprotein (N) gene variants specific to the SARS-CoV-2 virus, and then cleavage of a reporter molecule to produce a readable signal for the virus. A readable and positive result is generated only if both genes are detected to prevent any false positives resulting from related coronaviruses.^59,60^ Broughton et al. reported the development of a rapid, accurate, and easy-to-use technique based on CRISPR-Cas12 lateral flow assay for detection of SARS-CoV-2 virus from nasopharyngeal swab RNA extracts.^60^ The DETECTR system in the study generates a positive result when both E and N genes are detected, which makes the system accurate for SARS-CoV-2 detection when there are other viral respiratory infections.^60^

**Figure 4:**
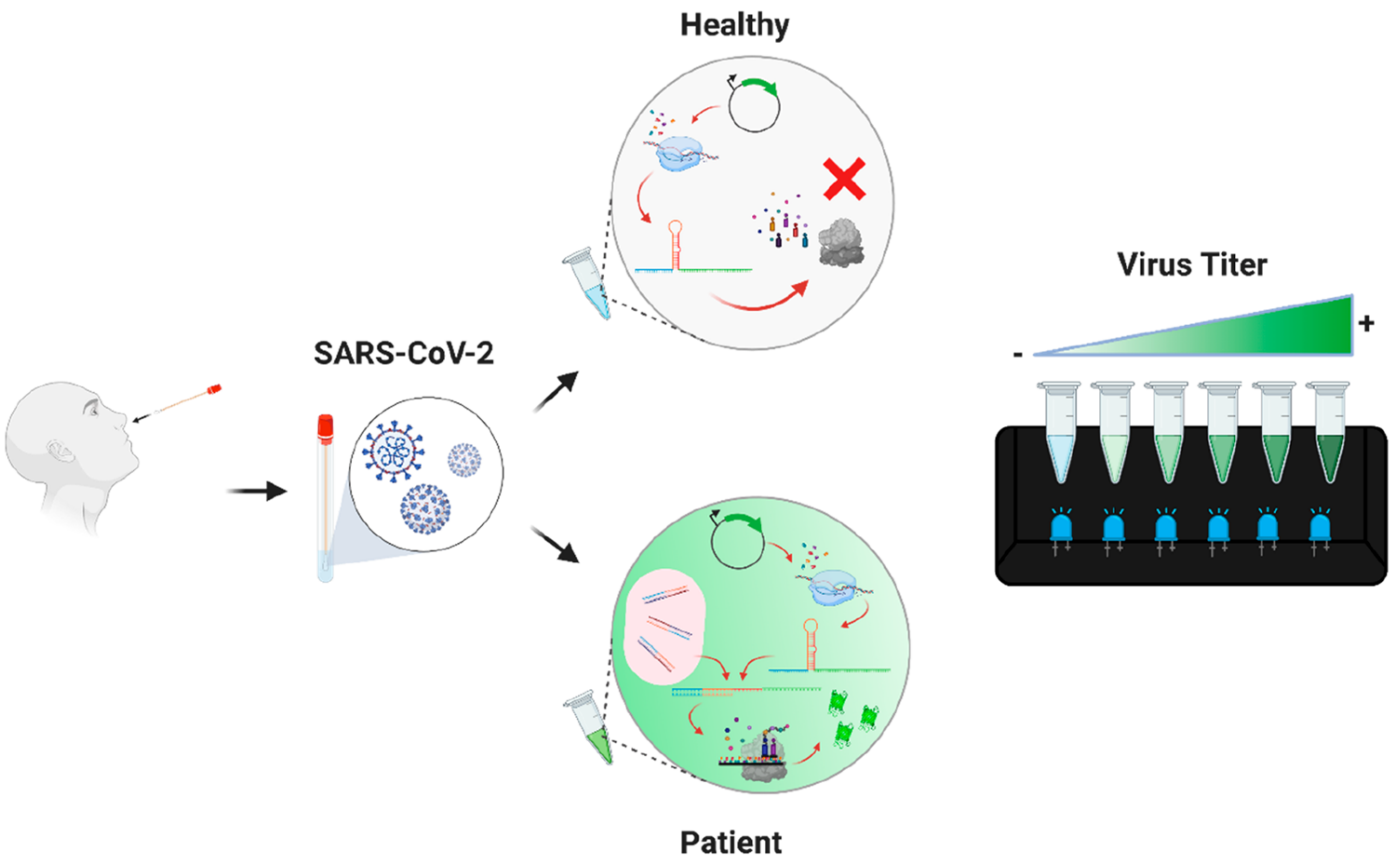
Schematic representation of the workflow of programmable toehold switch sensors. Viral RNA is isolated from patients’ swab samples. The fluorescence signal is observed when the sensors detect SARS-CoV-2-specific genomic regions.

The programmable RNA sensors are another promising in vitro synthetic biology approach for SARS-CoV-2 virus rapid detection and report that are easy and low-cost to develop. The riboregulatory toehold switches are a class of RNAs that can be used to trigger RNAs of interest and permit the translation of the reporter protein.^68^ The system has been proven to be versatile in detecting pathogenic viruses such as Zika and Ebola viruses, which make them of great potential to be utilized to develop a rapid and inexpensive POC detection method for the SARS-CoV-2 virus.^62,69^ With the cell-free transcription/translation (TXTL) technology, toehold-based sensors, as CRISPR-based techniques, are used to detect the presence of specific nucleic acid sequences with the output signal of a fluorescent protein or a colorimetric change.^63,69^ Koksaldi et al. successfully designed synthetic programmable toehold switch sensors to detect genomic regions specific to SARS-CoV-2 virus in which the presence of SARS-CoV-2-related genes triggers the translation of sfGFP mRNAs that can be monitored using a hand illuminator for the visibility of their toehold sensor responses (Figure 4).^63^ Such assays, when the sensitivity to certain pathogen is improved, have proven to be a promising technology as they are easily applicable with a decreased detection time pronounced as minutes and without the need of a full-scale laboratory environment, and an expert in the field.

In the fight against the current COVID-19 pandemic, scientists have analyzed millions of different protein sequences to find the most suitable candidates for a synthetic vaccine and peptidomimetic therapeutic design.^70,71^ In fact, some compounds have been successfully adapted, designed, and repurposed to be used as therapeutics. Many research laboratories and companies have undertaken drug and vaccine development to reduce the spread and restrict COVID-19 morbidity and mortality.

Research exploring COVID-19 drugs focusing on preventing either the crucial pathways for viral transmission or multiplication are of great importance, especially with the arising variants and slow pace of vaccination. Some studies demonstrate the potential of designed small proteins against the Spike protein of the SARS-CoV-2 virus. One such example is nanobodies to directly disable the SARS-CoV-2 coronavirus. The development of a novel synthetic nanobody, an antibody with a single domain, within bacteria or yeast, enables reduced cost for the production, therefore making them easier to reach for the developing world countries.^72,73^ Another advancement is the computer-designed miniproteins which have been shown to protect lab-grown human cells from SARS-CoV-2 virus by binding to the Spike protein and neutralizing the virus with great efficiency.^74^ The availability of an aerosolized delivery route of these molecules directly to the nasal and lung epithelia provides a distinctive potential therapeutic strategy against not only the COVID-19 pandemic but also many respiratory viral infections.^75,76^

Multiple strategies have been reported to generate SARS-CoV-2 vaccines, including DNA- and RNA-based vaccines, viral vector vaccines, inactivated virus vaccines, live-attenuated virus vaccines, and recombinant protein vaccines.^77^ The BioNTech/Pfizer and Moderna vaccines exploit a new vaccination technology, messenger (mRNA)-based vaccines.^78−80^ The logic is to deliver a lipid nanoparticle encapsulated synthetic version of the mRNA that a virus uses to build its infectious proteins, which will provoke an immune response upon an antigen presence; in this case it is the viral Spike protein that binds to ACE2 receptor of the host cell and produces neutralizing antibodies when the SARS-CoV-2 virus is present.^81^ Being very promising, this synthetic mRNA-based vaccine is the first FDA-approved COVID-19 vaccine.

Research exploring COVID-19 drugs focuses on preventing either the crucial pathways for viral transmission or multiplication. Antiviral synthetic drugs which were originally discovered for various other viral infections have been in clinical use with COVID-19 patients. Although no effective antiviral drug is currently available to treat COVID-19 or any other human coronavirus infections, the FDA has approved for a Phase III trial a synthetic biological drug, carrimycin, an antibacterial drug to be effectively used in the COVID-19 infection.^82,83^ It is a novel antibacterial and anti-inflammatory drug produced by genetically engineered *Streptomyces spiramyceticus* having a 4′′-O-isovaleryltransferase gene from *Streptomyces thermotolerant*.^82^ With this modification, carrimycin obtains more potent antibacterial activity. Repurposed carrimycin has been shown to inhibit postentry replication events, especially the synthesis of viral RNA without causing significant side effects in the treatment of severe COVID-19 patients.

## Conclusion and Perspectives

Addressing the threats of new and lethal pathogens requires accurate, reproducible techniques for better diagnostics tests, drug discovery, and therapy. Diagnosis of infectious diseases still heavily relies on conventional methods for detecting the presence of a pathogen, yet it has some limitations, such as the need of well-established and full-scale laboratories and qualified personnel, lacking standardized protocols, being time-consuming, and being more prone to produce false-negative and false-positive results; these assays are far from being reliable POC testings.^84,85^ Synthetic biology provides solutions to limitations of conventional diagnostics in the fight against deadly outbreaks by accurately and efficiently improving the techniques and POC testing to gain medical advantage in both industrialized and low-income countries. Being a multidisciplinary field, synthetic biology exploits advancements in both basic and applied research in genetics, microbiology, biochemistry, computer science, and engineering to program microorganisms that offer rapid, sensitive, specific, affordable, and noninvasive methods for infectious disease diagnostics and treatment.

Where bacterial and viral infections worldwide caused 90% of the outbreaks,^86^ the need for an effective therapy is so vital that many academic laboratories, physicians, and biotech companies have been performing tremendous efforts to design, develop, and readdress drug and vaccine candidates. All we can hope for our common effort is to be better prepared for future outbreaks, after we witnessed how the recent COVID-19 pandemic ran havoc around the whole world.
